# The genome sequence of a sawfly, *Macrophya alboannulata *(Costa, 1859)

**DOI:** 10.12688/wellcomeopenres.20897.1

**Published:** 2024-02-12

**Authors:** Steven Falk, Andrew Green, University of Oxford and Wytham Woods Genome Acquisition Lab, Darwin Tree of Life Barcoding collective, Wellcome Sanger Institute Tree of Life Management, Samples and Laboratory team, Wellcome Sanger Institute Scientific Operations: Sequencing Operations, Wellcome Sanger Institute Tree of Life Core Informatics team, Tree of Life Core Informatics collective, Darwin Tree of Life Consortium

**Affiliations:** 1 Independent researcher, Kenilworth, England, UK; 2 Sawfly Recording Scheme, Bedford, England, UK

**Keywords:** Macrophya alboannulata, sawfly, genome sequence, chromosomal, Hymenoptera

## Abstract

We present a genome assembly from an individual female *Macrophya alboannulata* (sawfly; Arthropoda; Insecta; Hymenoptera; Tenthredinidae). The genome sequence is 245.2 megabases in span. Most of the assembly is scaffolded into 8 chromosomal pseudomolecules. The mitochondrial genome has also been assembled and is 23.17 kilobases in length. Gene annotation of this assembly on Ensembl identified 24,359 protein coding genes.

## Species taxonomy

Eukaryota; Metazoa; Eumetazoa; Bilateria; Protostomia; Ecdysozoa; Panarthropoda; Arthropoda; Mandibulata; Pancrustacea; Hexapoda; Insecta; Dicondylia; Pterygota; Neoptera; Endopterygota; Hymenoptera; Tenthredinoidea; Tenthredinidae; Tenthredininae; *Macrophya*; *Macrophya punctumalbum* (Costa, 1859) (NCBI:txid1384894).

## Background

There are approaching 300 *Macrophya* species globally of which ten are present in Britain. The genus is characterised by elongate hind femora and coxae. *Macrophya alboannulata* (Costa, 1859) is within the subgenus *Macrophya*. In addition to the subgenera, several species groupings have been named and *M. alboannulata* is within the *Macrophya epinota* group together with *Macrophya albicincta* (Schrank, 1776) and *Macrophya ribis* (Schrank, 1781). Many *Macrophya* species are, as yet, unassigned to any subgenus.

*Macrophya alboannulata* was split from *M. albicincta* by Chevin (1975), and so its true historic status in Britain is not entirely clear. Some older *M. albicincta* records may have been uncritically, or erroneously, redetermined to *M. alboannulata* as the number of records is higher than might be expected. However, most recent records under the name *M. alboannulata* would seem to be named correctly ( Musgrove, 2023). Liston (1983) noted that *M. alboannulata* seemed to be found mostly in southern Britain, but there are two recent Scottish records.

*Macrophya alboannulata* is a relatively large (10–11 mm) *Macrophya* species. The insect is black, marked with white to varying degrees on the clypeus, labrum and postocellar region, the pronotum, legs and the margins of some abdominal tergites. Whilst similar to *M. albicincta* and *M. ribis*, *M. alboannulata* can be recognised in the field with careful examination. *Macrophya ribis* tends to be smaller and has dense course punctures around the frons and temples. The two remaining species can be separated by the colouring of the hind trochanter and trochantellus as described by Chevin (1975). In *M. albicincta* the hind trochanters are predominantly black and the trochantelli usually white with a black spot, whereas in *M. alboannulata* the hind trochanters are predominantly marked with white and the trochantelli never have a black spot. In males the labrum of *M. albicincta* is at least darkened at the lateral edges to mostly black, whereas in *M. alboannulata* the labrum is at most slightly infuscate at the lateral margins.

Most sawfly species are predatory, or feed on pollen or nectar, but the feeding habits of adult *M. alboannulata* are unclear. The larvae feed on elder ( *Sambucus nigra* L.), and are not considered a pest of agricultural or horticultural significance in Britain. The species is univoltine with adults on the wing from April to July. This female *M. alboannulata* from Wytham Woods, England was identified using Benson’s key ( Benson, 1952) with reference to Chevin’s identification characteristics.

Whilst some *Macrophya* species exhibit BIN sharing, *M. alboannulata* appears to be a well-defined species with barcoded specimens falling in a single cluster AAK6380 ( BOLD Systems, 2023). Knowledge of sawfly evolution will benefit from the comparative analysis of genomes from closely and distantly related species. This complete gene sequence will help our understanding of the phylogeny of this group.

## Genome sequence report

**Figure 1.**
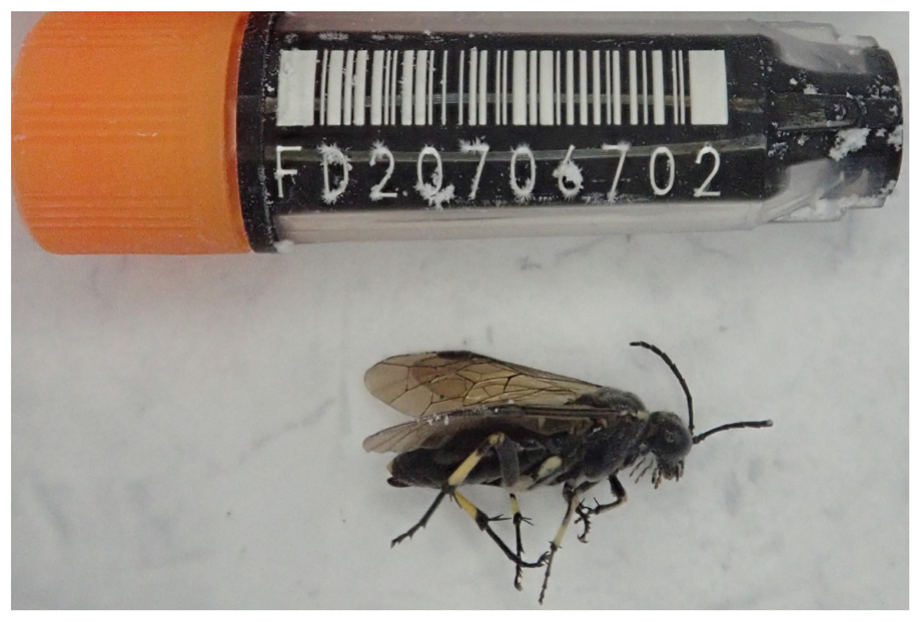
Photograph of the *Macrophya alboannulata* (iyMacAlbo1) specimen used for genome sequencing.

The genome was sequenced from one female *Macrophya alboannulata* ( Figure 1) collected from Wytham woods, Oxfordshire, UK (51.76, –1.33). A total of 101-fold coverage in Pacific Biosciences single-molecule HiFi long reads was generated. Primary assembly contigs were scaffolded with chromosome conformation Hi-C data. Manual assembly curation corrected 101 missing joins or mis-joins and removed 7 haplotypic duplications, reducing the assembly length by 1.16% and the scaffold number by 54.63%, and increasing the scaffold N50 by 110.89%.

**Figure 2.**
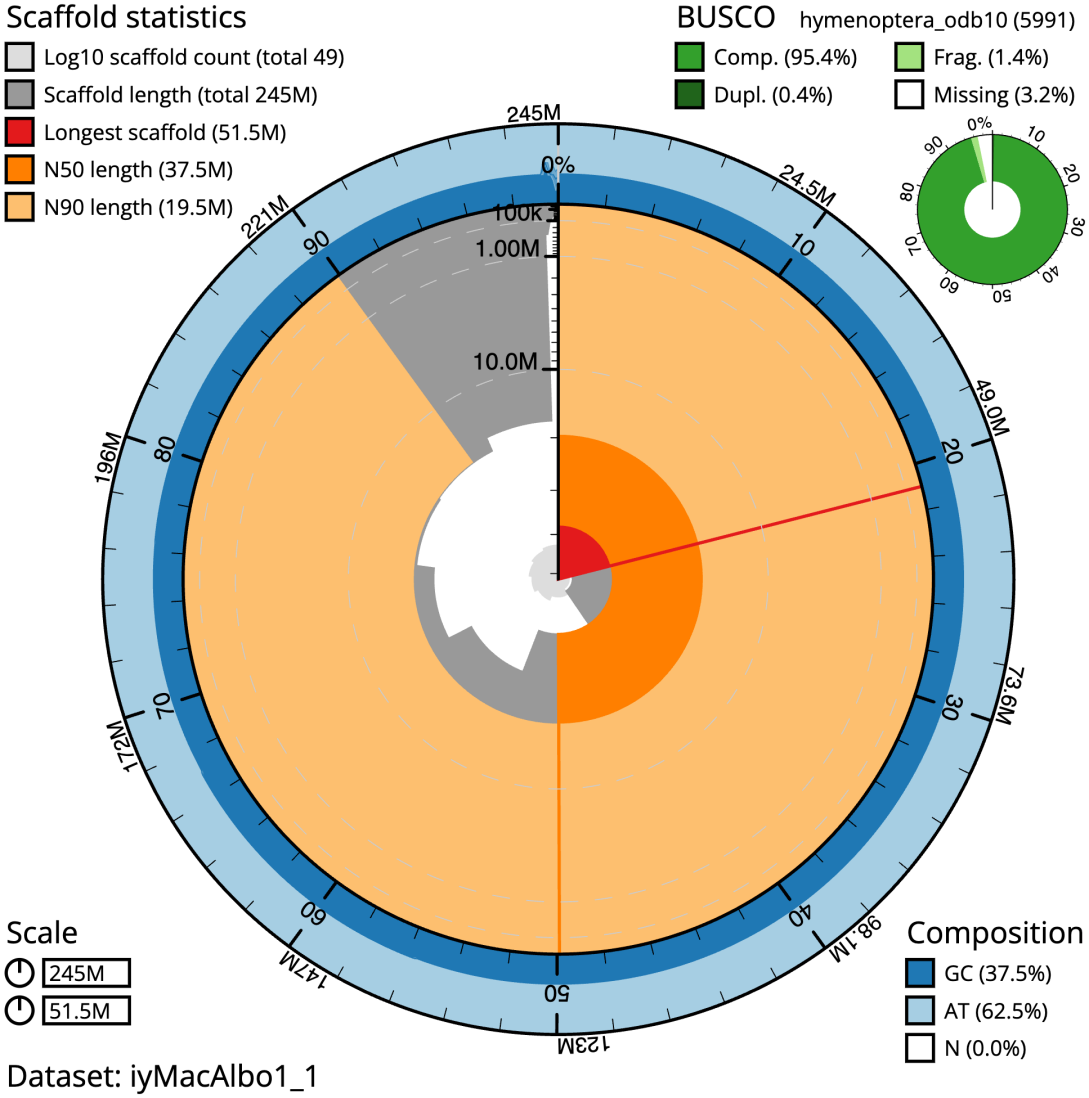
Genome assembly of *Macrophya alboannulata*, iyMacAlbo1.1: metrics. The BlobToolKit Snailplot shows N50 metrics and BUSCO gene completeness. The main plot is divided into 1,000 size-ordered bins around the circumference with each bin representing 0.1% of the 245,239,222 bp assembly. The distribution of scaffold lengths is shown in dark grey with the plot radius scaled to the longest scaffold present in the assembly (51,530,056 bp, shown in red). Orange and pale-orange arcs show the N50 and N90 scaffold lengths (37,453,143 and 19,476,270 bp), respectively. The pale grey spiral shows the cumulative scaffold count on a log scale with white scale lines showing successive orders of magnitude. The blue and pale-blue area around the outside of the plot shows the distribution of GC, AT and N percentages in the same bins as the inner plot. A summary of complete, fragmented, duplicated and missing BUSCO genes in the hymenoptera_odb10 set is shown in the top right. An interactive version of this figure is available at https://blobtoolkit.genomehubs.org/view/Macrophya%20alboannulata/dataset/iyMacAlbo1_1/snail.

**Figure 3.**
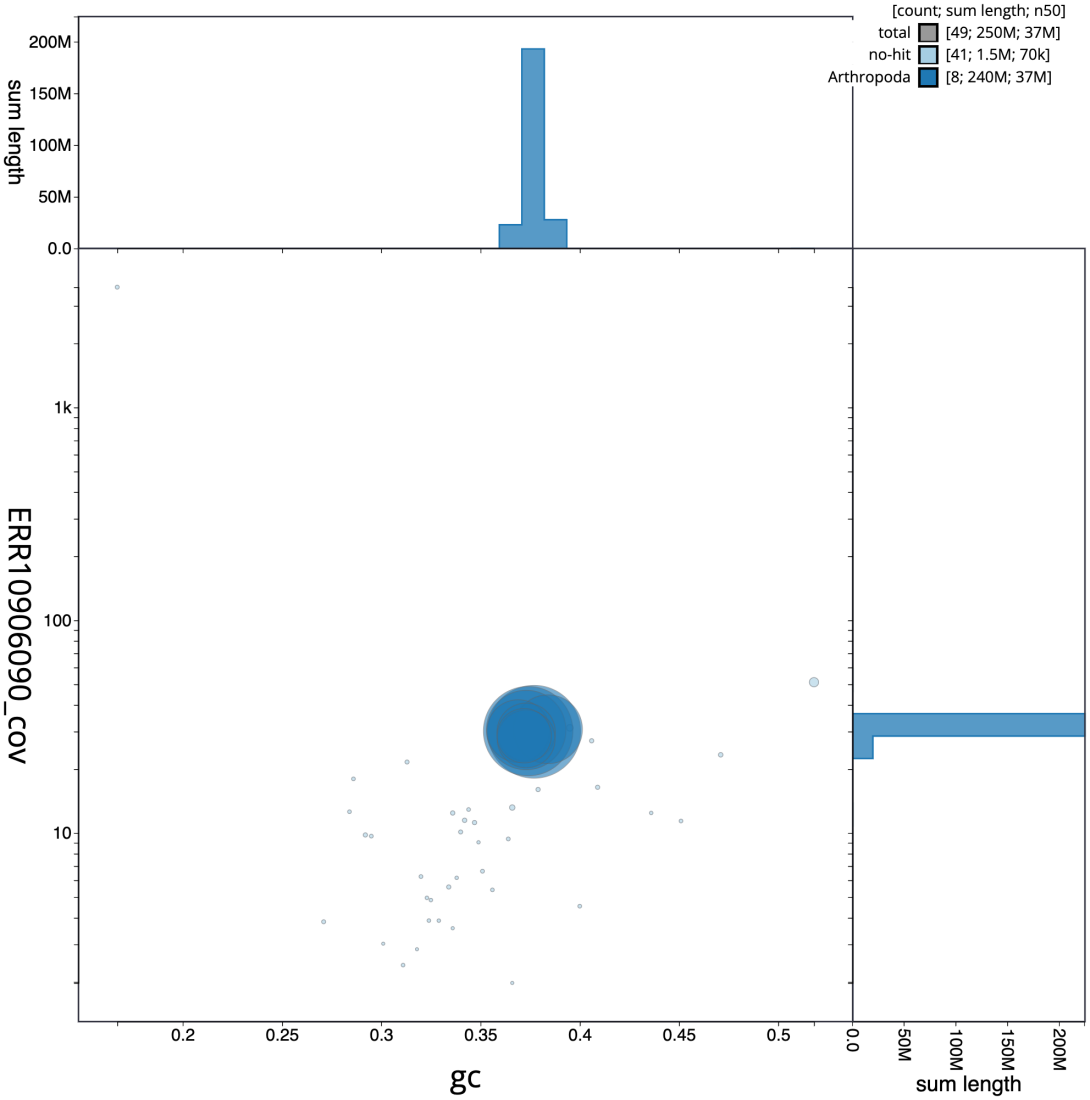
Genome assembly of *Macrophya alboannulata*, iyMacAlbo1.1: BlobToolKit GC-coverage plot. Scaffolds are coloured by phylum. Circles are sized in proportion to scaffold length. Histograms show the distribution of scaffold length sum along each axis. An interactive version of this figure is available at https://blobtoolkit.genomehubs.org/view/Macrophya%20alboannulata/dataset/iyMacAlbo1_1/blob.

**Figure 4.**
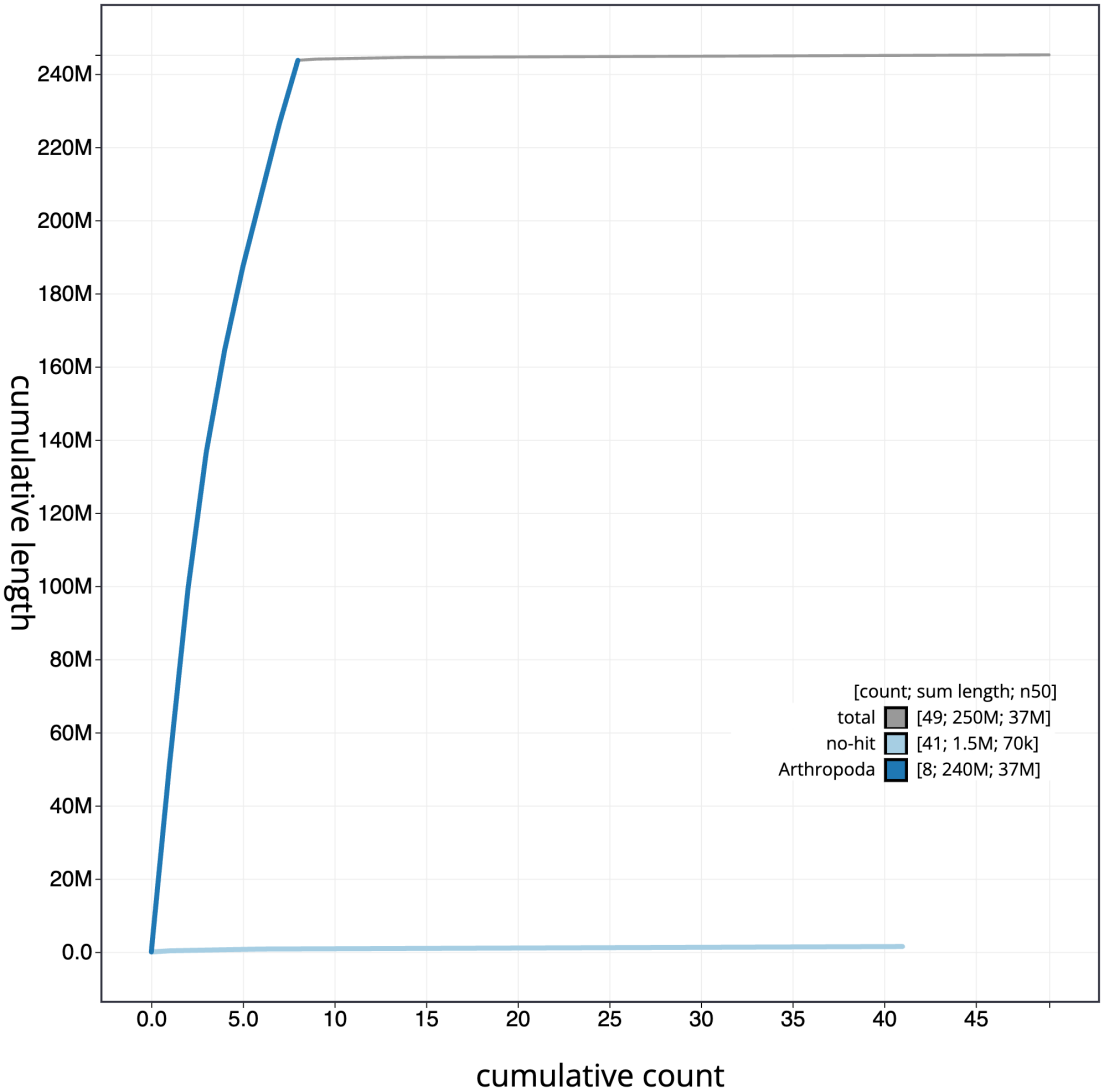
Genome assembly of *Macrophya alboannulata*, iyMacAlbo1.1: BlobToolKit cumulative sequence plot. The grey line shows cumulative length for all scaffolds. Coloured lines show cumulative lengths of scaffolds assigned to each phylum using the buscogenes taxrule. An interactive version of this figure is available at https://blobtoolkit.genomehubs.org/view/Macrophya%20alboannulata/dataset/iyMacAlbo1_1/cumulative.

**Figure 5.**
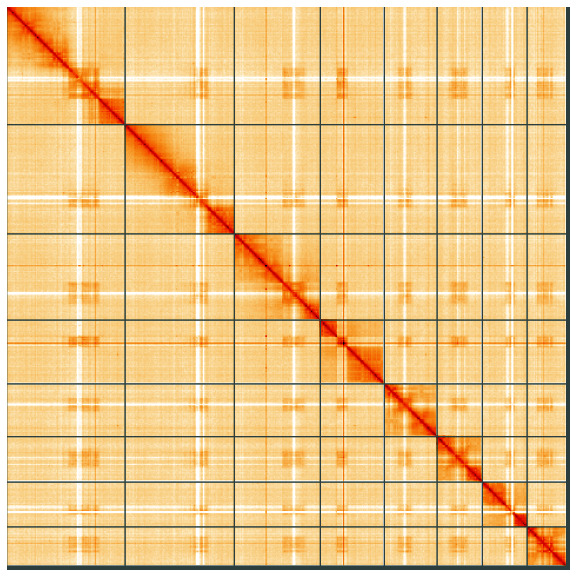
Genome assembly of *Macrophya alboannulata*, iyMacAlbo1.1: Hi-C contact map of the iyMacAlbo1.1 assembly, visualised using HiGlass. Chromosomes are shown in order of size from left to right and top to bottom. An interactive version of this figure may be viewed at https://genome-note-higlass.tol.sanger.ac.uk/l/?d=UQaGT2x2RDq3FRlYtdORtg.

The final assembly has a total length of 245.2 Mb in 48 sequence scaffolds with a scaffold N50 of 37.5 Mb ( Table 1). The snailplot in Figure 2 provides a summary of the assembly statistics, while the distribution of assembly scaffolds on GC proportion and coverage is shown in Figure 3. The cumulative assembly plot in Figure 4 shows curves for subsets of scaffolds assigned to different phyla. Most (99.38%) of the assembly sequence was assigned to 8 chromosomal-level scaffolds. Chromosome-scale scaffolds confirmed by the Hi-C data are named in order of size ( Figure 5; Table 2). While not fully phased, the assembly deposited is of one haplotype. Contigs corresponding to the second haplotype have also been deposited. The mitochondrial genome was also assembled and can be found as a contig within the multifasta file of the genome submission.

**Table 1.** Genome data for *Macrophya alboannulata*, iyMacAlbo1.1.

Project accession data		
Assembly identifier	iyMacAlbo1.1	
Species	*Macrophya alboannulata*	
Specimen	iyMacAlbo1	
NCBI taxonomy ID	1384894	
BioProject	PRJEB59942	
BioSample ID	SAMEA10167065	
Isolate information	iyMacAlbo1, female: thorax (DNA sequencing), head (Hi-C sequencing)	
Assembly metrics *		*Benchmark*
Consensus quality (QV)	55.9	*≥ 50*
*k*-mer completeness	99.99%	*≥ 95%*
BUSCO **	C:95.4%[S:95.0%,D:0.4%], F:1.4%,M:3.2%,n:5,991	*C ≥ 95%*
Percentage of assembly mapped to chromosomes	99.38%	*≥ 95%*
Sex chromosomes	-	*localised homologous pairs*
Organelles	Mitochondrial genome assembled	*complete single alleles*
Raw data accessions		
PacificBiosciences SEQUEL II	ERR10906090	
Hi-C Illumina	ERR10908619	
Genome assembly		
Assembly accession	GCA_949628255.1	
*Accession of alternate haplotype*	GCA_949628235.1	
Span (Mb)	245.2	
Number of contigs	465	
Contig N50 length (Mb)	1.2	
Number of scaffolds	48	
Scaffold N50 length (Mb)	37.5	
Longest scaffold (Mb)	51.5	
Genome annotation		
Number of protein-coding genes	24,359	
Number of gene transcripts	24,653	

* Assembly metric benchmarks are adapted from column VGP-2020 of “Table 1: Proposed standards and metrics for defining genome assembly quality” from ( Rhie *et al.*, 2021). ** BUSCO scores based on the hymenoptera_odb10 BUSCO set using v5.3.2. C = complete [S = single copy, D = duplicated], F = fragmented, M = missing, n = number of orthologues in comparison. A full set of BUSCO scores is available at https://blobtoolkit.genomehubs.org/view/Macrophya%20alboannulata/dataset/iyMacAlbo1_1/busco.

**Table 2.** Chromosomal pseudomolecules in the genome assembly of *Macrophya alboannulata*, iyMacAlbo1

INSDC accession	Chromosome	Length (Mb)	GC%
OX451206.1	1	51.53	37.5
OX451207.1	2	47.62	37.5
OX451208.1	3	37.45	37.5
OX451209.1	4	27.84	38.5
OX451210.1	5	22.93	37.0
OX451211.1	6	19.78	37.5
OX451212.1	7	19.48	37.5
OX451213.1	8	17.12	37.0
OX451214.1	MT	0.02	17.0

The estimated Quality Value (QV) of the final assembly is 55.9 with *k*-mer completeness of 99.99%, and the assembly has a BUSCO v5.3.2 completeness of 95.4% (single = 95.0%, duplicated = 0.4%), using the hymenoptera_odb10 reference set ( *n* = 5,991).

Metadata for specimens, barcode results, spectra estimates, sequencing runs, contaminants and pre-curation assembly statistics are given at https://links.tol.sanger.ac.uk/species/1384894.

## Genome annotation report

The *Macrophya alboannulata* genome assembly (GCA_949628255.1) was annotated using the Ensembl rapid annotation pipeline ( Table 1; https://rapid.ensembl.org/Macrophya_alboannulata_GCA_949628255.1/Info/Index?db=core). The resulting annotation includes 24,653 transcribed mRNAs from 24,359 protein-coding genes.

## Methods

### Sample acquisition and nucleic acid extraction

A female *Macrophya alboannulata* (specimen ID Ox001511, ToLID iyMacAlbo1) was netted in Wytham Woods, Oxfordshire (biological vice-county Berkshire), UK (latitude 51.76, longitude –1.33) on 2021-05-31. The specimen was collected and identified by Steven Falk (independent researcher) and then snap-frozen on dry ice.

Protocols developed by the Wellcome Sanger Institute (WSI) Tree of Life Core Laboratory have been deposited on protocols.io ( Denton *et al.*, 2023b). The workflow for high molecular weight (HMW) DNA extraction at the WSI includes a sequence of procedures: sample preparation; sample homogenisation, DNA extraction, fragmentation, and clean-up. The iyMacAlbo1 sample was weighed and dissected on dry ice ( Jay *et al.*, 2023), with tissue set aside for Hi-C sequencing. Tissue from the thorax was homogenised using a PowerMasher II tissue disruptor ( Denton *et al.*, 2023a). HMW DNA was extracted in the WSI Scientific Operations core using the Automated MagAttract v2 protocol ( Oatley *et al.*, 2023). HMW DNA was sheared into an average fragment size of 12–20 kb in a Megaruptor 3 system with speed setting 31 ( Bates *et al.*, 2023). Sheared DNA was purified by solid-phase reversible immobilisation ( Strickland *et al.*, 2023): in brief, the method employs a 1.8X ratio of AMPure PB beads to sample to eliminate shorter fragments and concentrate the DNA. The concentration of the sheared and purified DNA was assessed using a Nanodrop spectrophotometer and Qubit Fluorometer and Qubit dsDNA High Sensitivity Assay kit. Fragment size distribution was evaluated by running the sample on the FemtoPulse system.

### Sequencing

Pacific Biosciences HiFi circular consensus DNA sequencing libraries were constructed according to the manufacturers’ instructions. DNA sequencing was performed by the Scientific Operations core at the WSI on a Pacific Biosciences SEQUEL II instrument. Hi-C data were also generated from head tissue of iyMacAlbo1 using the Arima2 kit and sequenced on the Illumina NovaSeq 6000 instrument.

### Genome assembly, curation and evaluation

Assembly was carried out with Hifiasm ( Cheng *et al.*, 2021) and haplotypic duplication was identified and removed with purge_dups ( Guan *et al.*, 2020). The assembly was then scaffolded with Hi-C data ( Rao *et al.*, 2014) using YaHS ( Zhou *et al.*, 2023). The assembly was checked for contamination and corrected as described previously ( Howe *et al.*, 2021). Manual curation was performed using HiGlass ( Kerpedjiev *et al.*, 2018) and Pretext ( Harry, 2022). The mitochondrial genome was assembled using MitoHiFi ( Uliano-Silva *et al.*, 2023), which runs MitoFinder ( Allio *et al.*, 2020) or MITOS ( Bernt *et al.*, 2013) and uses these annotations to select the final mitochondrial contig and to ensure the general quality of the sequence.

A Hi-C map for the final assembly was produced using bwa-mem2 ( Vasimuddin *et al.*, 2019) in the Cooler file format ( Abdennur & Mirny, 2020). To assess the assembly metrics, the *k*-mer completeness and QV consensus quality values were calculated in Merqury ( Rhie *et al.*, 2020). This work was done using Nextflow ( Di Tommaso *et al.*, 2017) DSL2 pipelines “sanger-tol/readmapping” ( Surana *et al.*, 2023a) and “sanger-tol/genomenote” ( Surana *et al.*, 2023b). The genome was analysed within the BlobToolKit environment ( Challis *et al.*, 2020) and BUSCO scores ( Manni *et al.*, 2021; Simão *et al.*, 2015) were calculated.

Table 3 contains a list of relevant software tool versions and sources.

**Table 3 .** Software tools: versions and sources.

Software tool	Version	Source
BlobToolKit	4.2.1	https://github.com/blobtoolkit/blobtoolkit
BUSCO	5.3.2	https://gitlab.com/ezlab/busco
Hifiasm	0.16.1-r375	https://github.com/chhylp123/hifiasm
HiGlass	1.11.6	https://github.com/higlass/higlass
Merqury	MerquryFK	https://github.com/thegenemyers/MERQURY.FK
MitoHiFi	2	https://github.com/marcelauliano/MitoHiFi
PretextView	0.2	https://github.com/wtsi-hpag/PretextView
purge_dups	1.2.3	https://github.com/dfguan/purge_dups
sanger-tol/genomenote	v1.0	https://github.com/sanger-tol/genomenote
sanger-tol/readmapping	1.1.0	https://github.com/sanger-tol/readmapping/tree/1.1.0
YaHS	1.2a	https://github.com/c-zhou/yahs

### Genome annotation

The BRAKER2 pipeline ( Brůna *et al.*, 2021) was used in the default protein mode to generate annotation for the *Macrophya alboannulata* assembly (GCA_949628255.1) in Ensembl Rapid Release.

### Wellcome Sanger Institute – Legal and Governance

The materials that have contributed to this genome note have been supplied by a Darwin Tree of Life Partner. The submission of materials by a Darwin Tree of Life Partner is subject to the **‘Darwin Tree of Life Project Sampling Code of Practice’**, which can be found in full on the Darwin Tree of Life website here. By agreeing with and signing up to the Sampling Code of Practice, the Darwin Tree of Life Partner agrees they will meet the legal and ethical requirements and standards set out within this document in respect of all samples acquired for, and supplied to, the Darwin Tree of Life Project.

Further, the Wellcome Sanger Institute employs a process whereby due diligence is carried out proportionate to the nature of the materials themselves, and the circumstances under which they have been/are to be collected and provided for use. The purpose of this is to address and mitigate any potential legal and/or ethical implications of receipt and use of the materials as part of the research project, and to ensure that in doing so we align with best practice wherever possible. The overarching areas of consideration are:

- Ethical review of provenance and sourcing of the material
- Legality of collection, transfer and use (national and international)

Each transfer of samples is further undertaken according to a Research Collaboration Agreement or Material Transfer Agreement entered into by the Darwin Tree of Life Partner, Genome Research Limited (operating as the Wellcome Sanger Institute), and in some circumstances other Darwin Tree of Life collaborators.

## Data Availability

European Nucleotide Archive: *Macrophya alboannulata*. Accession number PRJEB59942; https://identifiers.org/ena.embl/PRJEB59942 ( Wellcome Sanger Institute, 2023). The genome sequence is released openly for reuse. The *Macrophya alboannulata* genome sequencing initiative is part of the Darwin Tree of Life (DToL) project. All raw sequence data and the assembly have been deposited in INSDC databases. Raw data and assembly accession identifiers are reported in Table 1.

## Author information

Members of the University of Oxford and Wytham Woods Genome Acquisition Lab are listed here: https://doi.org/10.5281/zenodo.7125292.

Members of the Darwin Tree of Life Barcoding collective are listed here: https://doi.org/10.5281/zenodo.4893703.

Members of the Wellcome Sanger Institute Tree of Life Management, Samples and Laboratory team are listed here: https://doi.org/10.5281/zenodo.10066175.

Members of Wellcome Sanger Institute Scientific Operations: Sequencing Operations are listed here: https://doi.org/10.5281/zenodo.10043364.

Members of the Wellcome Sanger Institute Tree of Life Core Informatics team are listed here: https://doi.org/10.5281/zenodo.10066637.

Members of the Tree of Life Core Informatics collective are listed here: https://doi.org/10.5281/zenodo.5013541.

Members of the Darwin Tree of Life Consortium are listed here: https://doi.org/10.5281/zenodo.4783558.
