# Supplementary material for: The genome sequence of a sawfly, Macrophya alboannulata (Costa, 1859)
Source: Wellcome Open Res. 2024 Feb 12;9:28. [Version 1] doi: 10.12688/wellcomeopenres.20897.1 (PMC11358680; doi:10.12688/wellcomeopenres.20897.1)
Supplement: Peer review history

# Reviewer report: Reviewer response for version 1

**DOI:** 10.21956/wellcomeopenres.23123.r94168

**Published:** 2024-08-28

**Authors:** Alexis L Sperling

**Affiliations:** 1 University of Cambridge, Cambridge, England, UK

Presented is the genome assembly of the sawfly, *Macrophya alboannulata*, which is endemic to Britain. This work is part of the Darwin Tree of Life Project which is working towards sequencing and assembling the genomes of eukaryotic organisms endemic to Britain and Ireland. This is an incredibly important project and this report is an excellent contribution to this effort.

 This work is scientifically sound and exceptionally well presented. All of the data that I would want to see is clearly presented in figures and tables of this report. The image of the actual female sawfly sequenced is an important detail that is often missed with genomics data in the literature. The remaining figures are very clear and beautifully present all of the relevant data for this effectively phased genome assembly. The snail plot in Figure 2 and the Hi-C contact map in Figure 5 clearly demonstrates how well this genome has been assembled.

 All of the methods are clearly explained within the text as well as within the methods section.

 This is a very good report that will be useful to anyone performing comparative genomics within this group of animals and for anyone wishing to research this sawfly specifically.

Are sufficient details of methods and materials provided to allow replication by others?

Yes

Is the rationale for creating the dataset(s) clearly described?

Yes

Are the datasets clearly presented in a useable and accessible format?

Yes

Are the protocols appropriate and is the work technically sound?

Yes

Reviewer Expertise:

Genomics, Diptera, parthenogenesis, genetics, crop science.

I confirm that I have read this submission and believe that I have an appropriate level of expertise to confirm that it is of an acceptable scientific standard.

# Reviewer report: Reviewer response for version 1

**DOI:** 10.21956/wellcomeopenres.23123.r74661

**Published:** 2024-05-25

**Authors:** Romain Gastineau

**Affiliations:** 1 University of Szczecin, Szczecin, Poland

The article 'The genome sequence of a sawfly, *Macrophya alboannulata *(Costa, 1859)' describes the obtention of a rather complete nuclear genome for a female specimen of species of Hymenoptera sampled in UK.

 The protocol is clearly explained and the depth of sequencing seem to have been correct enough as it allowed the authors to retrieve a ca. 250Mb genome in only 48 contigs.

 Authors are obvious native English speakers so the language quality of the article reflects this.

Are sufficient details of methods and materials provided to allow replication by others?

Yes

Is the rationale for creating the dataset(s) clearly described?

Yes

Are the datasets clearly presented in a useable and accessible format?

Yes

Are the protocols appropriate and is the work technically sound?

Yes

Reviewer Expertise:

Organellar genomes, taxonomy and phylogeny. Diatoms and Geoplanidae.

I confirm that I have read this submission and believe that I have an appropriate level of expertise to confirm that it is of an acceptable scientific standard.
